# Complete Genome Sequence of Enterotoxigenic Escherichia coli Myophage LL12

**DOI:** 10.1128/MRA.00675-19

**Published:** 2019-07-25

**Authors:** Denish Piya, Lauren Lessor, Mei Liu, Jason J. Gill

**Affiliations:** a Department of Biochemistry and Biophysics, Texas A&M University, College Station, Texas, USA; b Center for Phage Technology, Texas A&M University, College Station, Texas, USA; c Department of Animal Science, Texas A&M University, College Station, Texas, USA; Loyola University Chicago

## Abstract

This work describes the complete genome sequence of the virulent myophage LL12. The 136-kb LL12 genome is related to coliphage V5 and is a component in the prebiotic PreforPro. LL12 was isolated against enterotoxigenic Escherichia coli, which causes traveler’s diarrhea.

## ANNOUNCEMENT

Enterotoxigenic Escherichia coli (ETEC) strains can be distinguished from other E. coli pathotypes by the presence of enterotoxins which induce traveler’s diarrhea (TD), which is characterized by mild to severe watery diarrhea (1). Typically, TD self-resolves or may be treated with antibiotics, but due to the increase in the emergence of antibiotic resistance, alternative treatment approaches, such as phage therapy, have been suggested (2).

Phage LL12 was isolated from a municipal water treatment plant in College Station, TX, by growing a deidentified clinical ETEC isolate in the wastewater sample supplemented with LB broth (Difco) at 37°C with aeration. Upon isolation, LL12 was propagated using a nonpathogenic E. coli K-12 strain, DH5-alpha, by the soft-agar overlay method (3). Phage DNA was purified using a modified Wizard DNA purification kit (Promega) as described previously (4). Phage LL12 DNA was prepared for sequencing as part of a pooled indexed DNA library using the Illumina TruSeq nano low-throughput (LT) kit and sequenced by an Illumina HiSeq 2500 instrument as unpaired 100-base pair reads by sequencing by synthesis (SBS) V2 chemistry; 17,009,166 raw reads generated were quality controlled by FastQC (https://www.bioinformatics.babraham.ac.uk/projects/fastqc/) and assembled with Velvet 1.1 (5) into a single contig at 28.3-fold coverage. The contig was closed based on its circular assembly which produced an identical sequence at each end of the contig. Structural annotation was conducted using GLIMMER 3.0 (6) and MetaGeneAnnotator 1.0 (7), with tRNAs predicted by ARAGORN 2.36 (8); gene functions were predicted by InterProScan 5.15-54.0 (9), the NCBI Conserved Domains Database (10), TMHMM 2.0 (http://www.cbs.dtu.dk/services/TMHMM), BLASTp 2.2.8 (11), and HHpred 2.1 (12). Genome annotation was conducted using the Phage Galaxy (13) and WebApollo (14) instances hosted by the Center for Phage Technology (https://cpt.tamu.edu/), and all analyses were conducted using default parameters. Phages were imaged by transmission electron microscopy at the Texas A&M Microscopy and Imaging Center as previously described (15, 16).

The myophage LL12 has a genome of 136,026 bp, with a G+C content of 43.6%. It contains 213 predicted protein-coding genes and 7 tRNAs. Based on BLASTp (E value, ≤10^−5^), LL12 is most closely related to V5-like myophages, including rV5 (GenBank accession no. NC_011041; 206 shared proteins) and ϕAPCEc02 (KR698074; 204 shared proteins). LL12 is also related to the phage phi92 (NC_023693), with 48 common proteins. An analysis of the Illumina reads by PhageTerm (17) suggests the presence of a nonpermuted terminal redundancy of 459 bp spanning bases 104,966 to 105,424 in the genome sequence as deposited. This predicted terminal repeat is located in a noncoding region between two convergent transcripts. Fifty LL12-encoded proteins could be assigned putative functions, including the capsid protein (gp59), portal protein (gp63), and large terminase subunit (gp64). The components for tail morphogenesis, including baseplate (gp35) and multiple predicted tail fiber proteins (gp27, gp29, gp32, gp33, gp36, gp41, and gp42), were identified. The genes encoding the large terminase subunit (gp64) and DNA polymerase (gp212) are disrupted by predicted intron sequences. The genes responsible for phage lysis, including the phage endolysin (gp90), i-spanin (gp66), and o-spanin (gp65), were identifiable, but the phage holin could not be positively identified.

### Data availability.

The annotated phage genome sequence is deposited in NCBI GenBank under accession no. MH491969. Associated BioProject, SRA, and BioSample accession numbers are PRJNA222858, SRR9113738, and SAMN11840293, respectively.
